# Parental Knowledge and Awareness of the First Permanent Molar

**DOI:** 10.5005/jp-journals-10005-1544

**Published:** 2018-10-01

**Authors:** Alireza Heydari, Mahdi Shahrabi, Maryam Shafizadeh, Elnaz A Anaraki, Mostafa Aref

**Affiliations:** 1 Assistant Professor, Department of Pediatric Dentistry, Dental School, Tehran University of Medical Sciences, Tehran, Islamic Republic of Iran; 2 Associate Professor, Department of Pediatric Dentistry, Dental School, Tehran University of Medical Sciences, Tehran, Islamic Republic of Iran; 3 Assistant Professor, Department of Pediatric Dentistry, School of Dentistry, Ilam University of Medical Sciences, Ilam, Islamic Republic of Iran; 4 Postgraduate Student, Department of Pediatric Dentistry, Dental School, Tehran University of Medical Sciences, Tehran, Islamic Republic of Iran; 5 Dentist, Department of Pediatric Dentistry, Dental School, Tehran University of Medical Sciences, Tehran, Islamic Republic of Iran

**Keywords:** Deciduous, DMF index, dentition; Education, Permanent, Prenatal.

## Abstract

**Background and Aim:**

This study aimed to assess the awareness of parents about the presence of first permanent molar and its effect on the health of this tooth.

**Materials and methods:**

This cross-sectional research has been done on 250 elementary school students which were 6 to 8-year-old boys and their parents in Tehran city. Sampling was done randomly. The questionnaires were filled out by the parents, and then tooth condition was registered in a visit formfor each student separately. Next, the related children’s decayed missing filled teeth (DMFT) was determined and recorded. The mean value was calculated. Data were analyzed using the statistical package for the social sciences (SPSS) via a generalized estimating equation.

**Results:**

There was a significant relationship between parental awareness of being permanent of the first permanent molar and DMFT (p < 0.05).

**Conclusion:**

Socio-economical factors like parental education can have an effect on oral health condition.

**How to cite this article:** Heydari A, Shahrabi M, Shafizadeh M, Anaraki EA, Aref M, Parental Knowledge and Awareness of the First Permanent Molar. Int J Clin Pediatr Dent, 2018;11(5):382-385.

## INTRODUCTION

Based on a report published in the American Department of Health in May 2000, it has been suggested that the tooth decay is the most common chronic disease in childhood.^1^ Which has been affecting many people everywhere in the world and It has many problems and costs. Dental caries is a multifactorial infectious disease that requires a host, nutrient culture and bacteria which produce acid. The bacterial plaque in the mouth feeds from substrates such as fermented foods and produces acid causing tooth decay.^2^

The activity of dental caries can be increased by using sugar, a type that will stick to the tooth surface, andif the frequency of using this type of sugar increases, it will increase the chance of tooth decay.^3^

An inappropriate diet of the child and unbalanced consumption of chocolate and sweets, in addition the inability to observe the oral health properly, cause tooth decay. Since children are not aware of the importance of oral hygiene and are not able to brush their teeth properly, somain oral and dental care is their parent’s responsibility.

The fact that children acquire food habits, oral health and dental care from their parents, it causes tooth decay to become an environmental disease.^4^

The first permanent molar due to early time of eruption (between the ages of 6-7),^5^ the presence of grooves and cavities on the occlusal surface and other factors initially related to the early age of young children, including the lack of proper sanitation and unbalanced consumption of sugar content, is more predisposed to caries.^3^

Sometimes, parents think that the first permanent molar is a deciduous tooth and instead of restoring it, they extract the tooth and deprives the child of the right to permanent teeth in the future, and they make up a lot of problems.

## MATERIALS AND METHODS

This cross-sectional study was conducted on the parents of 6-8-year-old students in Tehran in 2012. From this population, multistage sampling (cluster and random sampling, proportional to volume) 250 people were selected.

Ten districts of 22 districts of Tehran were selected based on their geographic location to determine the random samples of the community.

The data collection tool was a questionnaire and an examination. The questionnaire’s validity was assessed by reviewing the literature and under the supervision of the professors in the expert panel of the questionnaire. By using a pre-test, a sample of 20 parents of students and a questionnaire for measuring the reliability of the questionnaire were used to prepare the final questionnaire. After completing the final questionnaire by parents, the children were examined by a researcher with a headlamp with a mirror and probe.

Quantitative variables were reported as mean, and standard deviation and qualitative variables were reported as number and percentage. Generalized estimating equation with exchangeable matrix and a linear model was used to assess the correlation between variables and DMFT. The p < 0.05 was considered statistically significant.

Questionnaire questions were in 4 sections: 1-Parents and child specifications (12 questions). 2-Oral and dentition health status of a child (3 questions). 3-Knowledge about permanent teeth (4 questions). 4-Knowledge of oral and dentition health (1 question).

It should be noted that 267 questionnaires were collected and all of them were included in the study.

## RESULTS

With regard to the correlation of DMFT with quantitative variables, the following results were obtained:

*Father’s education:* There was a significant relationship between DMFT and father’s education. (p < 0.043). The lowest DMFT was in children whose fathers had a bachelor’s degree or higher.

*Mother’s education:* There was not a significant relationship between DMFT and mother’s education. (p < 0.161)

*The frequency of brushing tooth by the child:* There wasn’t a significant relationship between DMFT and the frequency of brushing tooth by the child. (0.615).

*Living district:* There was a significant relationship between the living district and DMFT (p < 0.003). Lowest DMFT in this study were those children who were studying in District,^3^ and most DMFT were children who were studying in District.^2^

*Family income:* There was a significant relationship between family income and DMFT. (p < 0.045). The lowest DMFT was for families with income more than 300 dollars per month.

With regard to the correlation of parental awareness of the eruption time of the first permanent molar^6^ with quantitative variables, the following results were obtained:

*Father’s education:* There was a significant relationship between parental awareness of the eruption time of the first permanent molar^6^ and father’s education. (p < 0.014).

*Mother’s education:* There was not a significant relationship between parental awareness of the eruption time of the first permanent molar^6^ and mother’s education. (p < 0.079)

*The frequency of brushing tooth by the child:* There was not a significant relationship between parental awareness of the eruption time of the first permanent molar^6^ and the frequency of brushing tooth by the child. (0.478)

*Living district:* There was a significant relationship between the living district and parental awareness of the eruption time of the first permanent molar^6^ (p < 0.501).

*Family income:* There was a significant relationship between family income and parental awareness of the eruption time of the first permanent molar^6^ (p < 0.342).

According to the study, parents who did not know the existence of permanent teeth in their children’s mouth, their children had an average of four permanent teeth.

Parents who were not aware of the presence of permanent teeth (just first molar) in their children’s mouth, their children had an average of two permanent teeth 6 in their mouths.

The result of this is that many parents are not aware of the presence of permanent teeth (first molar) in their children’s mouth.

## DISCUSSION

The profession of dentistry has been changed in many ways. By changing the needs of humans during the time of this profession, extraction has been replaced by filling and nowadays prevention is the best way. Failure to observe oral hygiene increases the amount of decay in the teeth. As stated in this thesis, the permanent tooth is formed when children cannot do proper oral health activities. Therefore, it is the responsibility of the parents to observe and do hygiene of their mouth. Unfortunately, the lack of awareness of many of them leads to serious damage to the child’s dental system. According to the statistics in the thesis, dentists should be considered as one of the most important sources to increase the awareness of the parents about this tooth and its importance. This is important when the dentists’ attitude of treatment to prevention is changed. Increasing parental awareness about the risk of decay factors and the importance of first permanent molar can be an important step in maintaining the health of the child’s dental system.

Ravera et al.^6^ stated that there is a significant relationship between the level of parenting education and the dental status of children and its along with our study. Akpabio et al.^7^announced that mothers who had studied more years were more aware of oral hygiene issues. What is certain is that mothers’ education, like fathers, can affect their awareness and improve the oral health of children. Vanobberge et al.^8^ stated that the risk of increased caries in children is significantly related to the lowering of their parents’ job level. Goettems et al.^9^ stated that boys have higher DMFT in low-income families, and children with high-quality mothers have better dmft status than others. Cristina et al.^10^ reported that parents’ awareness was significantly related to their economic situation. De Rue et al.^11^ stated that living conditions had a significant relationship with oral hygiene behavior. Also, there was a meaningful relationship between the children’s living area and DMFT inour study too. It seems reasonable that DMFT decreases when oral hygiene increases and this can be achieved by increasing knowledge. Akpabio et al.^7^ suggested that high-income households were more aware of dental protection and oral hygiene behaviors, like our results, with an increase in the level of income, we see a better oral condition. Low-income families were less likely to follow health care. Borges et al.^12^ said that the prevalence of caries among children living in families with lower income and lower education was higher and mentioned that the outbreak of dental caries had been linked with access to the dentist. In the present study, the majority of parents who obtained oral health information from a dentist often had a baccalaureate, or higher education and these parents were the most who refer to the dentist every 6 months. Consequently, according to the results of this study, it can be verified that children who are more in contact with a dentist have a better oral condition. Kowash et al.^13^ stated that dental health education on mothers can have a significant effect on preventing the development of caries in children. In the present study, there is a significant relationship between the parent’s education and the frequency of tooth-brushing of the child. (p-value < 0.05) As in the group of children who sometimes brush their tooth, the level of education of parents is diploma or lower than that like our study; this article showed the importance of the effect of parental education on the parental awareness of the oral health of the child.

It should be noted that the inclusion of female students in research can have important implications in the outcome of the study, so Poutanen et al.^14^ carried out a study. He concluded that parent-related factors (including awareness, economics, etc.) have a different impact on dental health in both girls and boys. Among the boys, the father’s job level and among girls, the behavior and knowledge of parents have affected their dental health. It was also stated in this study that the role of parents is very important on the development of dental health in children. Therefore, according to the results of this study and related studies, parents’ knowledge about oral hygiene can have a significant effect on oral health and promote more and more healthy teeth, including permanent teeth.

## CONCLUSION

Parental awareness of being permanent of the first permanent molar^6^ was significantly correlated with DMFT, and socio-economical factors like parent’s education can have an effect on oral health condition.
